# High-Quality Draft Genome Sequence of *Vagococcus lutrae* Strain LBD1, Isolated from the Largemouth Bass *Micropterus salmoides*

**DOI:** 10.1128/genomeA.01087-13

**Published:** 2013-12-26

**Authors:** François Lebreton, Michael D. Valentino, Lucile B. Duncan, Qiandong Zeng, Abigail Manson Mcguire, Ashlee M. Earl, Michael S. Gilmore

**Affiliations:** Departments of Ophthalmology, Microbiology, and Immunobiology, Harvard Medical School, Massachusetts Eye and Ear Infirmary, Boston, Massachusetts, USAa; The Broad Institute, Cambridge, Massachusetts, USAb

## Abstract

Vagococci are usually isolated from marine hosts and occasionally from endodontic infections. Using 16S rRNA gene comparison, the closest relatives are members of the genera *Enterococcus* and *Carnobacterium*. A draft sequence of *Vagococcus lutrae* was generated to clarify the relationship of *Vagococcus* to these and other related low-G+C Gram-positive bacteria.

## GENOME ANNOUNCEMENT

The bacterial genus *Vagococcus* was proposed in 1989 for Gram-positive, catalase-negative, motile, coccus-shaped bacteria that react with Lancefield group N antisera (1). Phylogenetic trees based on 16S rRNA gene sequences place *Vagococcus* adjacent to the genera *Enterococcus* and *Carnobacterium* (2). The genus *Vagococcus* currently consists of eight species (*V. fluvialis, V. salmoninarum, V. lutrae, V. fessus, V. carniphilus, V. elongatus*, *V. penaei*, and *V. acidifermentans*) (1–8).

Most representatives of *Vagococcus* have been isolated from aquatic environments, suggesting that members of this genus have traits optimized for existence and survival in marine habitats (1–8). *V. fluvialis* strains have been suggested as a promising candidate probiotic for aquaculture, a critical economic activity practiced worldwide (9). Interestingly, strains of this genus have also been isolated from patients receiving endodontic treatment for periradicular lesions (10).

In this study, we sampled the intestine of a largemouth bass (*Micropterus salmoides*) that was caught in the wild in Maine. Following outgrowth on bile esculin azide agar, we isolated a strain of *V. lutrae* named LBD1. This strain was subjected to whole-genome sequencing and constitutes the first report of a genome of the species *V. lutrae* and the genus *Vagococcus.*

Genomic DNA was isolated with a DNeasy kit (Qiagen, Valencia, CA) and was quantified by a Qubit fluorometric assay (Invitrogen, Carlsbad, CA). The paired-end library (2 × 250 bp) was prepared using a Nextera XT DNA sample preparation kit (Illumina, San Diego, CA). The quality and quantity of the library DNA fragments were measured on an Agilent Technologies 2100 Bioanalyzer (Santa Clara, CA). Sequencing was carried out on the Illumina MiSeq personal sequencer platform at the Massachusetts Eye and Ear Infirmary (MEEI) Ocular Genomics Institute (Boston, MA). CLC Genomics Workbench version 4.9 software (CLC bio, Cambridge, MA) was used for *de novo* assembly on an i7 Intel dual-core workstation. For *V. lutrae* LBD1, 7.48 million paired-end reads were collected. The average coverage of the 1.83-Mb LBD1 assembled genome (21 scaffolds; scaffold N_50_, 81.28 kb) was 720×.

Protein-coding genes were predicted with Prodigal (11) and filtered to remove genes with >70% overlap with tRNAs or rRNAs, which were identified using tRNAscan-SE (12) and RNAmmer (13), respectively. The gene product names were assigned based on top BLAST hits against the SwissProt protein database and a protein family profile search against the TIGRfam equivalogs, followed by top BLAST hits to KEGG protein sequences. For *V. lutrae* LBD1, we identified 1,736 protein-coding genes (48% with “hypothetical protein” as the gene product name), 3 rRNA genes (one each for 5S, 16S, and 23S), and 49 tRNAs (19 amino acids with tRNA-Asn missing, probably in the contig gap regions). Additional analyses performed included Pfam (14), TIGRfam (15), KEGG (16), COG (17), GO (18), and TMHMM (19) analyses.

The availability of this genome sequence begins to illuminate the roles of vagococci as members of the microbiome of fish and other marine animals and may aid future studies related to the aquaculture industry and/or human medicine.

### Nucleotide sequence accession numbers.

This whole-genome project has been deposited at DDBJ/EMBL/GenBank under accession no. AYSH00000000. The version of *V. lutrae* LBD1 described in this paper is version AYSH01000000.
